# PIWI-Interacting RNA-004800 Is Regulated by S1P Receptor Signaling Pathway to Keep Myeloma Cell Survival

**DOI:** 10.3389/fonc.2020.00438

**Published:** 2020-04-15

**Authors:** Huanxin Ma, Huihan Wang, Fei Tian, Yuan Zhong, Zhuogang Liu, Aijun Liao

**Affiliations:** Department of Hematology, Shengjing Hospital of China Medical University, Shenyang, China

**Keywords:** piRNA, multiple myeloma, S1PR, PI3K/Akt/mTOR, cell survival

## Abstract

PIWI-interacting RNA (piRNA) is a kind of non-coding single stranded RNA which plays major roles in epigenetic expressions, genome rearrangement, and regulation of gene and protein. Because piRNAs are abnormally expressed in various cancers, they can be used as novel biomarkers and therapeutic targets. However, the roles of piRNAs in cancer cell growth and survival are not well-known. Here, we are the first to provide evidence that PIWI-interacting RNA-004800 (piR-004800) is overexpressed in both exosomes from multiple myeloma (MM) patients' bone marrow supernatant and primary MM cells. The expression level of piR-004800 is positively correlated with the stages of MM, according to the international staging system (ISS). In MM cell lines, downregulation of piR-004800 induced apoptosis and autophagic cell death. This was accompanied by *in vitro* and *in vivo* inhibition of cell proliferation. Our previous study shows that sphingosine-1-phosphate receptor (S1PR) signaling pathway plays a crucial part in MM cell proliferation. In this study, we find that S1PR signaling pathway can regulate the PI3K/Akt/mTOR pathway through control of piR-004800 expressions. Taken together our data supports an oncogenic role for piR-004800 in MM, which sheds insight into a new mechanism that may lead to therapeutic targets in MM, an incurable plasma cell neoplasm.

## Introduction

Multiple myeloma (MM) is the second most common hematological malignancy. As a plasma cell malignancy affecting the bone marrow, MM patients display an average 5-year survival rate of about 50% (1). Although MM patients' survival rate has been significantly improved with new therapeutics, a cure remains elusive. Specifically, the increases in drug resistance necessitates further exploration of MM pathogenesis.

Extensive evidence showed that exosomes play critical roles in the pathophysiological processes in tumor, such as immune antigen presentation, tumor growth and migration, and tissue injury repair (2, 3). As small extracellular vesicles (EVs), exosomes mediate local and systemic communication between cells by transferring bioactive substances, such as proteins, DNA, and RNAs. In exosomes, piRNAs account for 20–30% of total RNA. piRNAs are non-coding RNAs (ncRNAs) with a length of 24–32 nucleotides, and they are vital to maintain functions of germ and stem cells, by keeping genetic stability in germlines and through regulating epigenetics (4, 5). Because of these, research efforts have begun to explore the relationship between piRNA and tumor development. Existing studies show that piRNA was differentially expressed in stomach, lung, liver, and other solid tumors (6). In fact, piRNAs are significantly correlated with disease prognosis; they are linked to the occurrence, development, invasion, and metastasis of tumors. In MM, the contribution of piRNA to disease pathogenesis is not well-understood. One piRNA, piRNA-823, is shown to play the role in tumor formation by effect on DNA methylation and bone marrow microenvironment (7–9). This suggests a generalizable role for piRNAs in MM pathogenesis yet few have been identified. Here, we report the identification of piR-004800 as a possible oncogene of MM. Significantly expressed in MM, we show that piR-004800 is regulated by an upstream metabolic signaling that is dysregulated in MM, the S1PR signaling pathway.

S1P, a biologically active metabolite of sphingolipids, is included in many biologic processes. It specifically binds to and activates five G-protein coupled S1P receptors (S1PRs) Each S1PR can couple to different G-protein subunits (G_i/o_, G_q_, G_12/13_), but all S1PRs can couple with G_i/o_ protein (10). Through regulation of diverse downstream signaling pathway, S1PR signaling pathway affects various molecular and cellular events. Crucially, PI3K/AKT/mTOR plays a major role in the tumor cells' survival and proliferation, and it's reported to be regulated by S1PR signaling pathway (11). Abnormal activity in this pathway leads to malignant transformation, migration, adhesion, angiogenesis, apoptosis, and autophagy of cancer cells (12). Taken together, these suggest an oncogenic role for piR-004800.

Here, we report that S1PR signaling pathway regulates PI3K/AKT/mTOR signaling pathway through piR-004800 to promote MM pathogenesis. We will explore the effect of piR-004800 differential expression on MM cell survival and associated mechanisms.

## Materials and Methods

### Exosomal RNA Extraction and Sequencing Library Generation

The method to extract exosomal RNA and generate sequencing library was reported previously (13). Briefly the exosomal RNA was extracted by the miRNeasy Mini kit (Qiagen, Valencia, CA). Two nanogram exosomal RNA was used to constructed the small RNA libraries. Index libraries were equally pooled for sequencing (Illumina HiSeq4000 platform). After cleaning the initial data, we mapped sequences between 18 and 30 nt in length to reference genomes and other sRNA databases. The Transcripts Per Kilobase Million (TPM) is used to calculate the small RNA expression level. We used DEGseq, NOIseq, ExpDiff methods to screen differentially expressed sRNAs (DESs). Novel piRNAs were identified from sequencing data by piano. When scores ≥2, the predicted piRNAs were considered significant.

### Human Samples

Bone marrow supernatant was obtained from17 healthy donors and 66 newly diagnosed MM patients in Shengjing Hospital. Primary MM cells were obtained from 18 healthy donors and 29 newly diagnosed MM patients in Shengjing Hospital. We got the written informed consents from all patients and healthy donors. The medical ethics committee of Shengjing Hospital approved these study. Ficoll–Hypaque gradient centrifugation was used to isolate mononuclear cells. And then CD138+ microbead selection (MiltenyiBiotec, Auburn, CA, USA) was used to select the plasma cells.

### Antibodies and Reagents

Anti-Mcl-1 (1:1,000), anti-BAD (1:500), anti-Bcl-2 (1:1,000), anti-Bcl-XL (1:500), anti-cleaved caspase-3 (1:500), anti-Stat3 (1:1,000), anti-LC3B (1:1,500), anti-beclin1 (1:1,000), and anti-β-actin (1:1,000) antibodies were purchased from Signalway antibody (College Park, Maryland, USA). Anti-Akt (1:1,000), anti-p-Akt (1:1,000), anti-p- anti-mTOR (1:1,000), mTOR (1:1,000) antibodies were bought from Cell Signaling Technology Inc. (Danvers, MA, USA). Anti-P62 (1:1,000) was purchased from Proteintech Group Inc. (Wuhan, China) S1P was bought from Sigma-Aldrich (Cat. S9666, St. Louis, MO, USA). FTY720 was bought from Cayman Chemical Company (Ann Arbor, MI, USA).

### Exosomes Isolation and NanoSight Tracking Analysis

Exosomes from bone marrow supernatant were obtained by using the Total Exosome Isolation Kit (from plasma) (Invitrogen, Carlsbad, CA, USA). Exosomes from the cell culture supernatant were obtained as reported (14). Nanoparticle tracking analysis (NTA) at VivaCell Biosciences with ZetaView PMX 110 (Particle Metrix, Meerbusch, Germany) was used to measure the exosome particle size and concentration. The data was analyzed by corresponding software ZetaView 8.04.02.

### Cell Lines and Cell Culture

MM cell lines RPMI8226 and U266 were gifts from The First Bethune Hospital of Jilin University. The cells were cultured in RPMI-1640 medium (Cellgro, Mediatech, USA) containing 10% fetal bovine serum (Gibco, Thermo Fisher Scientific Inc., USA) in a 37°C, 5% CO_2_ conventional cell culture incubator.

### Transfection

The hsa-piRNA-004800 antagomir (antagomir-4800) with chemical modification was used to down-regulate piRNA-004800 expression in MM cells. The sequence of antagomir-4800 is: 5′-UUCGAGCCGGAUUCGAACCAGCGACCUAAGGA-3′. Chemically modified hsa-piRNA-004800 agomir (agomir-4800) was used to up-regulate piRNA-004800 expression. The sequence of agomir- 004800 is: sense 5′-UCCUUAGGUCGCUGGUUCGAAUCCGGCUCGAA-3′, antisense 5′-CGAGCCGGAUUCGAACCAGCGACCUAAGGAUU-3′. In our study, siRNA specific for CDC42 (siRNA-CDC42) was used to suppress CDC42. The sequence of siRNA-CDC42 is: sense 5′-GUGGAGUGUUCUGCACUUATT-3′, antisense 5′-UAAGUGCAGAACACUCCACTT-3′ (GenePharma, Shanghai, China). Cells were transfected with antagomir, agomir, or siRNA by using Lipofectamine 3000 (Invitrogen, Carlsbad, CA, USA). And the final concentration was 100 nM. Quantitative real-time PCR (qRT-PCR) was used to validate the efficiency of transfection.

### Cell Viability Assay

After the indicated treatments, 1 × 10^4^ cells were planted in each well in 96-well-plates. Cells were processed with MTS kit (Promega, USA), reading the absorbance at 490 nm wavelength by a 96-well-multiscanner autoreader (BioTek, USA).

### Apoptosis Assay

The indicated cells were processed with PE Annexin V Apoptosis Detection kit (BD Bioscience, San Diego, CA, USA). Then the cells were tested by flow cytometry (FACScalibur, BD Biosciences, San Diego, CA, USA). Using the FlowJo software to analyze these data.

### Quantitative Real-Time PCR

RNA from samples was extracted by using the RNAiso Plus Kit (Takara, Dalian, China). Non-coding small RNA was transcribed into cDNA and quantified by Hairpin-it^TM^ microRNA and U6 snRNA Normalization RT-PCR Quantitation Kit (GenePharma, Shanghai, China). Complementary cDNA of mRNA was obtained with PrimeScript RT reagent Kit (Takara, Dalian, China) and then used as template to quantify mRNA levels by SYBR Green RT-PCR Kit (Takara, Dalian, China); ABI 7500 FAST Real Time PCR System (Applied Biosystems, Foster City, CA, USA) was used to do PCR reaction. All the primers were purchased from GenePharma (Shanghai, China). The primers' sequence: human piR-004800 forward 5′-GGGTCCTTAGGTCGCTGGTTC-3′, reverse 5′-TATGGTTGTTCACGACTCCTTCAC-3′; human CDC42 forward 5′-CGTGACCTGAAGGCTGTCAA-3′, reverse 5′-ACACACCTGCGGCTCTTCTT-3′.

### Western Blot Analysis

This method was performed as previously described (15). The SDS-PAGE was used to separate 30–50 μg protein. Next, we transferred the protein to polyvinylidene fluoride membranes (PVDF membranes). After being blocked, selected primary antibodies were used to incubate with the membranes overnight at 4°C. Before incubated with secondary antibody, the membrane was washed three times. The signal was visualized by enhanced chemiluminescence.

### Tumor Xenograft Model Establishment

Four to five weeks female BALB/C nude mice (Beijing Hua Fukang Bioscience Company, Beijing, China) were placed and monitored in an environment without pathogen. All about animal studies were approved by the Research Ethics Committee of China Medial University. 1 × 10^7^ RPMI8226 cells were suited in 50 μl serum-free RPMI-1640 medium and 50 μl BD Matrigel™ Basement Membrane Matrix (BD, Bioscience, San Diego, CA, USA), then injected subcutaneously into nude mice. About 12 days after injection, animal treatment was started after a significant tumor was detected. Mice were randomly divided into two groups of six mice and injected with 15 μg (15 mg/kg) antagomir-4800 or antagomir-NC into the tumor per mouse. The mice were treated by the intratumoral delivery therapy every 3 days and in total for 5 times. We monitored the growth of tumor every 3 days for approximately 3 weeks. The formula: V = 0.5 × a × b^2^ was used to calculate the tumor volume. The longest and shortest tumor diameters were, respectively, represented by a and b. Finally, we killed the mice and collected the tumor. The collected tumors were divided into two parts. One parts was storied in −80°C for RNA and protein extraction, another part was fixed in formalin for immunohistochemistry.

### Immunohistochemistry

This method was performed as described previously (16): Tumors were fixed and paraffin-embedded. 2.5 μm tumor sections were dewaxed, rehydrated, endogenous peroxidase removed and antigen retrieval. Then the tumor sections were incubated with the selected primary antibodies: anti ki67, anti-Mcl-1, anti-Bcl-2, and anti-cleaved caspase 3 (Signalway antibody, College Park, Maryland, USA). Before mounting, tumor sections were counterstained and dehydrated.

### Statistical Analysis

In this study, we used the GraphPad software v7.0 (www.graphpad.com) to perform the statistical analysis and graph generation. Two groups' comparisons were performed using the Mann Whitney test, or paired/unpaired two-tailed Student's *t*-test. For all analyses, *p* <0.05 was considered statistically significant.

## Results

### piR-004800 Was Overexpressed in MM Exosome and Cells

**Figure 1:**
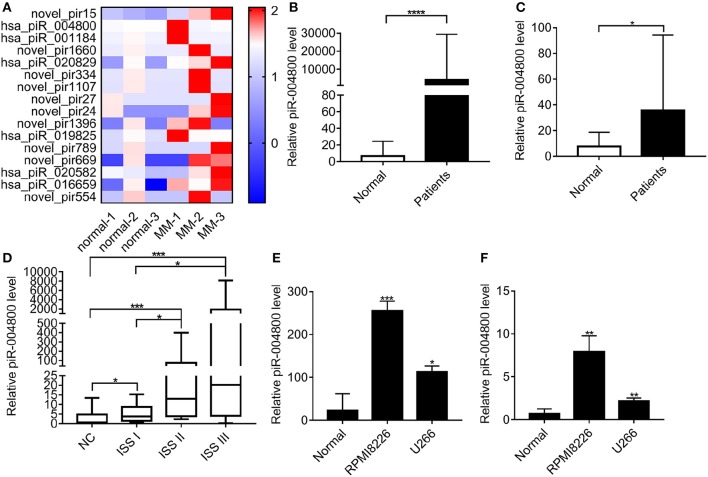
piR-004800 was highly expressed in MM patients and MM cell lines. **(A)** The heatmap for the differentially expressed piRNAs between the exosomes from normal and MM bone marrow supernatant. **(B)** Expression levels of piR-004800 in exosomes from bone marrow supernatant in MM patients (*n* = 56) and healthy donors (*n* = 17) were tested by qRT-PCR. **(C)** piR-004800 expression levels in CD138^+^ cells from MM patients (*n* = 29) and in bone marrow mononuclear cells from healthy donors (*n* = 18) were tested by qRT-PCR. **(D)** The expression of piR-004800 in MM patients with different ISS stages. ISS I (*n* = 11); ISS II (*n* = 15); ISS III (*n* = 30). **(E)** piR-004800 expression levels in MM cell lines and normal bone marrow mononuclear cells were tested by qRT-PCR. **(F)** piR-004800 expression levels in MM cell lines' exosomes and exosomes from bone marrow supernatant in healthy donors were tested by qRT-PCR. *P < 0.05, **P < 0.01, ***P < 0.001, and ****P < 0.0001.

We carried out small non-coding RNA sequencing in 6 exosome RNA samples (3 healthy donors and 3 MM patients) derived from bone marrow supernatant. Through this approach, the heatmap was established for 16 most highly expressed piRNAs (Figure 1A). To verify the top 3 highly expressed piRNAs, we analyzed the exosomes from additional MM patients (*N* = 56) and healthy donors (*n* = 17). From this, we found that piR-004800 is the most significantly expressed piRNA (Figure 1B). Analyzing primary MM cells and cell lines compared to bone marrow mononuclear cells from normal donors, we found that piR-004800 expression was significantly higher (Figure 1C). We run agarose gel electrophoresis to evaluate piRNA expression and molecular weight in the samples of MM patients and healthy controls (Supplemental Figure 1). The expression of piR-004800 in exosomes was positively correlated with that in MM cells from the same patients (Supplemental Figure 2). This leads us to inquire if the levels of piR-004800 are relative to the clinical stage of MM patients. We categorized MM patients based on disease progression according to the International Staging System (ISS): ISS I, ISS II, and ISS III. Compared to normal, ISS I, and ISS II groups, the average expression level of piR-004800 in ISS III patients was significantly higher (Figure 1D). We speculated that upregulation of piR-004800 correlates with MM progression.

### piR-004800 Modulated Proliferation and Apoptosis in MM Cells

**Figure 2:**
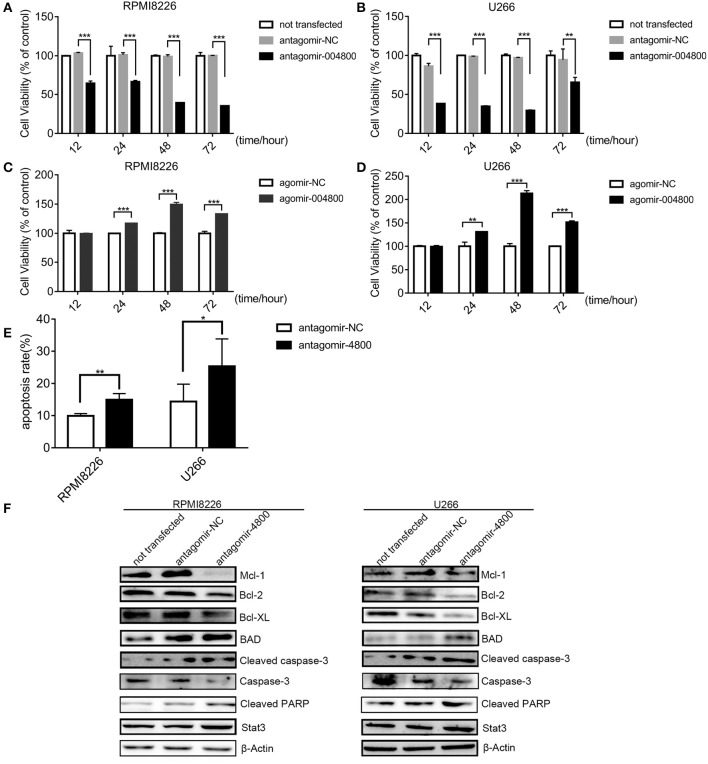
piR-004800 modulated proliferation and apoptosis in MM cells. **(A,B)** RPMI8226 and U266 cells were transfected with antagomir-NC, antagomir-4800, then the cells were harvested in 12, 24, 48, and 72 h; MTS assays was used to assess the cell viability. **(C,D)** RPMI8226 and U266 cells were transfected with agomir-NC, and agomir-4800, then the cells were harvested in 12, 24, 48, and 72 h; MTS assays was used to assess the cell viability. Averaged values ±sd. from three independent experiments are plotted. **(E)** RPMI8226 and U266 cells were transfected with antagomir-NC or antagomir-4800 and harvested after 48 h, then stained with PE Annexin V/7-AAD and analyzed by flow cytometry. **(F)** RPMI8226 and U266 cells were transfected with antagomir-NC or antagomir-4800 and harvested after 48 h, then Western blot analysis were explored. *P < 0.05, **P < 0.01, and ***P < 0.001.

Because piR-004800 was overexpressed in MM cell lines (Figures 1E,F), we next sought to characterize its biological functions in MM. Using antagomir-004800 to downregulate piR-004800, we observed significant time- and dose-dependent suppression of cell viability of RPMI8226 and U266 cell. In contrast, overexpression of piR-004800 had the reverse effects (Figures 2A–D). Using Annexin V/7-AAD flow cytometry analysis, we determined that the apoptosis rates of MM cells were significantly up-regulated in antagomir-004800 group compared to that in the antagomir-NC group (Figure 2E, Supplemental Figure 3). We also detected the apoptosis-related proteins. Specifically, the downregulation of piR-004800 decreases the expression of anti-apoptotic proteins Bcl-2, Bcl-XL, Mcl-1, and total caspase-3; Conversely, this increases the expression of pro-apoptotic proteins BAD, cleaved PARP, and cleaved caspase-3 (Figure 2F). However, this has no effect on Stat3.

### Downregulation of piR-004800 Induced Autophagic Death in MM Cells

**Figure 3:**
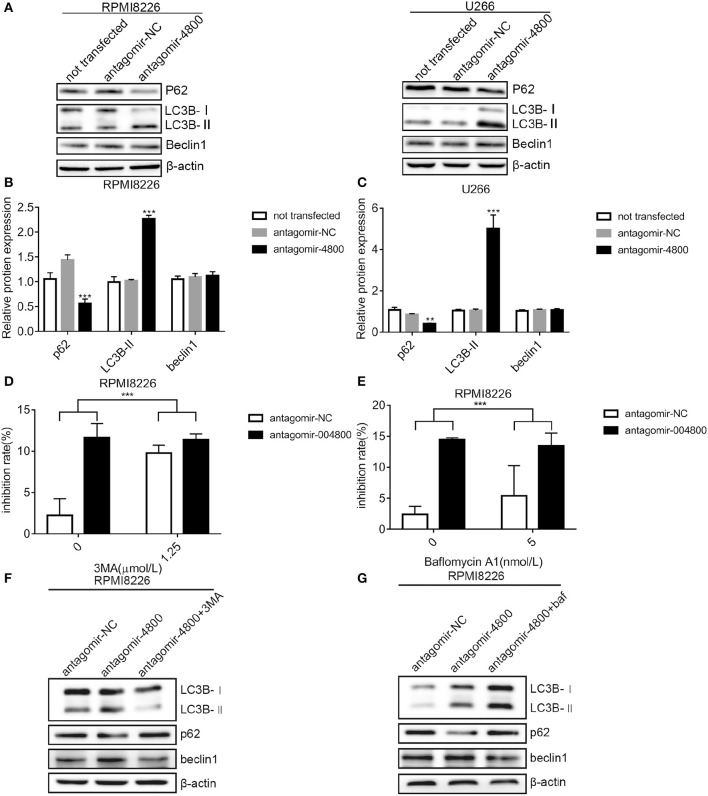
Downregulation of piR-004800 induced autophagic death in MM cells. **(A–C)** RPMI8226 and U266 cells were transfected with antagomir-NC or antagomir-4800, and harvested 48 h later, then Western blot analysis was explored. **(D)** RPMI8226 cells were transfected with antagomir-NC or antagomir-4800, 48 h later the cells were treated with 3MA for 24 h, then cell inhibition rate was assessed by MTS assays. **(E)** RPMI8226 cells were transfected with antagomir-NC or antagomir-4800, then 48 h later the cells were treated with Bafilomycin A1 for 24 h, and cell inhibition rate was tested by MTS assays. **(F,G)** The transfected RPMI8226 cells with antagomir-NC or antagomir-4800 were treated with 3MA or Bafilomycin A1, for 24 h, then the cells were harvested for Western blot analysis. **P < 0.01 and ***P < 0.001.

Autophagic death is a form of programmed cell death. We wanted to understand if piR-004800 had any effect on the autophagy of MM cells. Checking for markers of autophagy after piR-004800 knockdowns, our result showed that levels of LC3B-II was increased and p62 was decreased (Figures 3A–C). This indicated that down regulation of piR-004800 could induce autophagy in MM cells. To explore the effect of autophagy induced by down-regulation of piR-004800 on MM cell death, RPMI8226 cells with down-regulation of piR-004800 were treated with two autophagy inhibitors 3-methyladenine (3MA) and Bafilomycin A1. By MTS assay, it showed that autophagy inhibitors rescued cell death induced by piR-004800 knockdown (Figures 3D–G). The similar result was also got from U266 cells (Supplemental Figure 4). This result indicated that piR-004800 downregulation induces autophagy which could lead to cell death in MM cells.

### piR-004800 Was Regulated by S1PR Signaling Pathway in MM Cells

**Figure 4:**
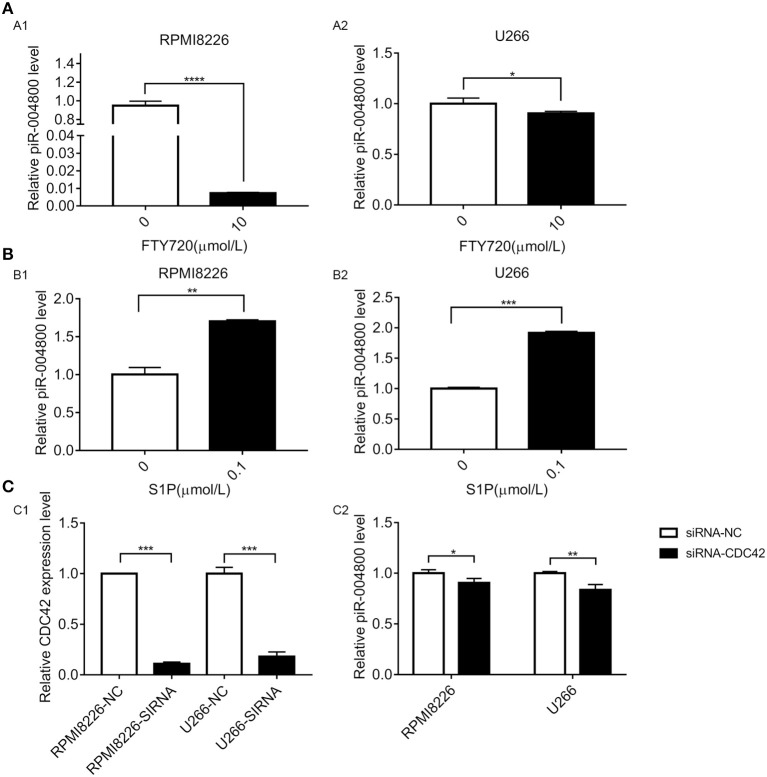
S1PR signaling pathway regulated the expression of piR-004800 in MM cells. **(A)** 10 μmol/L FTY720 treated RPMI8226 and U266 cells for 24 h, the expression of piR-004800 was assessed by qRT-PCR. **(B)** RPMI8226 and U266 cells were treated with 0.1 μmol/L S1P for 24 h, then the expression of piR-004800 was assessed by qRT-PCR. **(C1)** siRNA knockdown efficiency of CDC42 in two MM cell lines. **(C2)** Two MM cell lines were transfected with siRNA-NC and siRNA-CDC42, the expression of piR-004800 was analyzed by qRT-PCR in 48 h. *P < 0.05, **P < 0.01, ***P < 0.001, and ****P < 0.0001.

Our previous study found that activation of S1PR signaling pathway could promote survival of MM cell (15). Additionally, previous studies show that S1PR pathway could regulate the expression of small RNAs. Therefore, we wanted to determine whether S1PR pathway could regulate piR-004800 in MM cells. To inhibit or activate this pathway, we treated RPMI8226 and U266 cells with S1PR inhibitor FTY720 or activator S1P. FTY720 decreased the expression of piR-004800 while S1P increased its expression (Figures 4A,B). We previously showed that S1P5R, which couples with G_i_ and G_12/13_ is the most highly expressed receptor in U266 cells. Our study also showed the downstream targets of S1P5R were Rac1/PAK3 and CDC42/PAK3 (15). These drive the sequential activation of the PI3K/Akt/mTOR and Stat3/bcl-2 pathways. Using siRNA targeted to CDC42, we observed a concomitant decrease in the expression of piR-004800 in MM cells (Figure 4C). This suggests that piR-004800 was regulated by the S1PR signaling pathway.

### S1PR Signaling Pathway Regulated PI3K/Akt/mTOR Pathway Through piR-004800 in MM Cells

**Figure 5:**
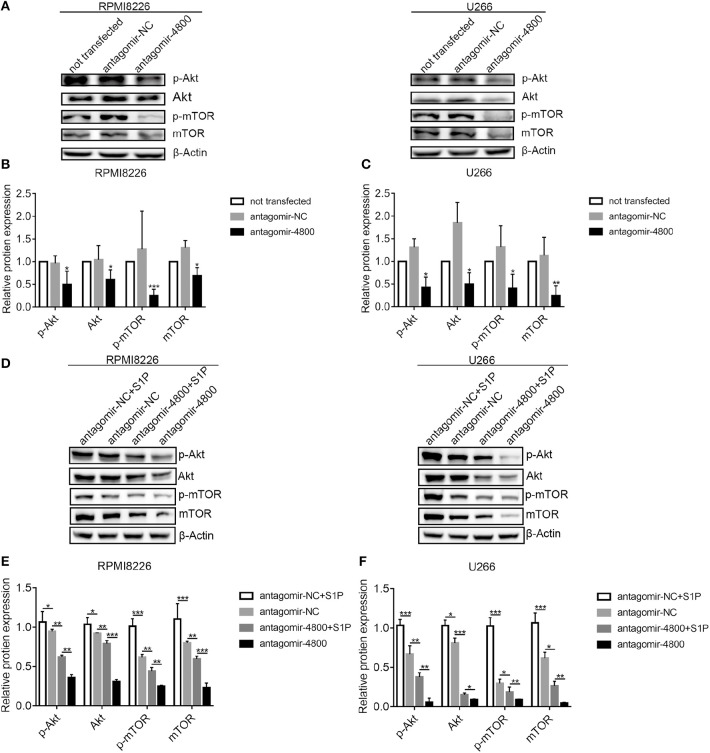
S1PR signaling pathway regulated PI3K/Akt/mTOR through piR-004800 in MM cells. **(A)** RPMI8226 and U266 cells were transfected with antagomir-NC or antagomir-4800, 48 h later Western blot analysis was explored. **(D)** RPMI8226 and U266 cells were transfected with antagomir-NC or antagomir-4800, 48 h later the cells were treated with S1P for 24 h, then Western blot was explored. **(B,C,E,F)** Relatively quantitative results was determined by Image J and shown as histogram. *P < 0.05, **P < 0.01, and ***P < 0.001.

Because the inhibition of the S1PR signaling could downregulate PI3K/Akt/mTOR pathway (15), we therefore explored whether piR-004800 could regulate PI3K/Akt/mTOR. Through inhibition of piR-004800, we observed significantly reduced expressions of p-Akt, Akt, p-mTOR, and mTOR in MM cells (Figures 5A–C). To further clarify if S1PR signaling pathway regulated PI3K/Akt/mTOR through piR-004800, we treated piR-004800 downregulated MM cells with S1P for 24 h. We found that the expressions of Akt, p-Akt, mTOR, and p-mTOR could be partly rescued (Figures 5D–F). This result indicated that S1PR signaling pathway could regulate PI3K/Akt/mTOR pathway through piR-004800.

### piR-004800 Promote MM Proliferation in Tumor Xenograft Model

**Figure 6:**
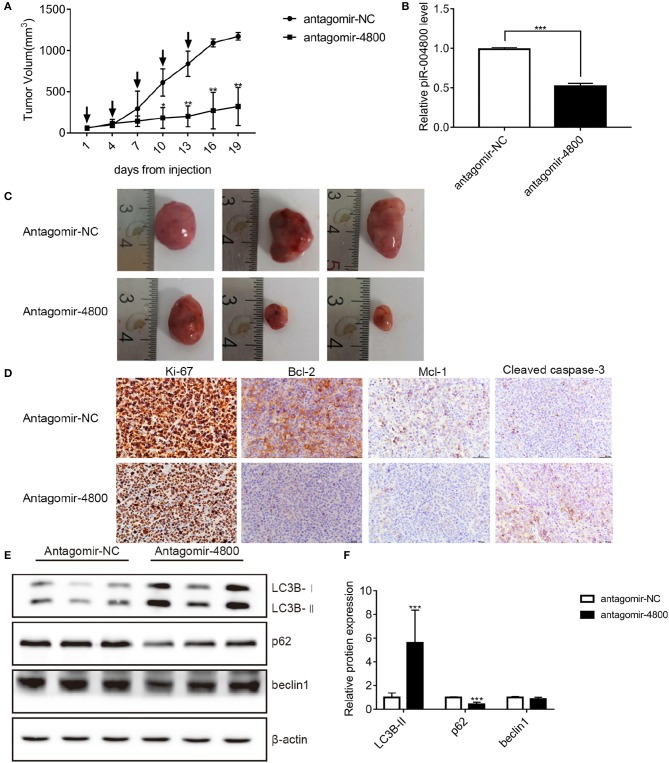
Effects of piR-004800 on the growth of RPMI8226 xenografts in mice. Antigomir-NC or antigomir-4800 was injected to intratumorally to treat the accessible subcutaneous xenografts every 3 days (indicated by arrows). A total of five injections were performed. **(A)** The change of tumor volumes which were measured every 3 days. **(B)** The levels of piR-004800 were analyzed by qRT-PCR using total RNA taken from xenografts. **(C)** The volume of the tumors with the treatment of antagomir-NC or antagomir-4800. **(D)** Tumor xenografts' typical immunohistochemical staining of selected markers (original magnification ×400). **(E,F)** The tumor cells were isolated from the xenografts and the protein was extracted from the cells, then Western blot assay was explored. *P < 0.05, **P < 0.01, and ***P < 0.001.

Finally, we wanted to further identify the *in vivo* biological function of piR-004800. We injected 1 × 10^7^ RPMI8226 cells subcutaneously into nude mice. Tumors developed in these mice 3 weeks after injection. We then treated the mice with injection of 15 μg antagomir-4800 or 15 μg antagomir-NC every 3 days for 2 weeks. Monitoring for tumor volume, the tumor growth in mice was significantly inhibited treated with antagomir-004800 (Figures 6A–C). Finally, immunohistochemical staining and Western blot were performed on tumor tissues. In antagomir-4800 group compared with that in antagomir-NC group, the expressions of Ki-67, Bcl-2, and Mcl-1 which related to cell proliferation was decreased while the expression of cleaved caspase-3 was increased (Figure 6D). The Western blot showed that the expression of p62 was decreased while the expression of LC3B-II was increased in antagomir-4800 group compared with that in antagomir-NC group (Figures 6E,F), which indicated the induced autophagy by knocking down of piR-004800. These results were consistent with the results of our *in vitro* experiments.

## Discussion

piRNA can play both an agonistic and antagonistic role in the development of cancer: the precise outcome is determined by tissue specificity. In lung cancer, piR-55490 inhibits cell growth by binding to the 3′UTR region of mTOR to induce the degradation of mTOR (17). piRNA-823 shows similar tumor suppressive properties (18). However, piRNA-823 is expressed at a higher level in MM, promoting tumorigenesis through DNA methylation (7). Finally, the oncogenic effect of certain differentially expressed piRNAs can be phenocopied across multiple cancers. For example, piRNA-651 has been shown to be associated with gastric, lung, colon and breast cancers (18–21). Taken together, these studies indicated potential of piRNA as a disease marker, yet its functions are not well-defined.

To further investigate the function of small RNA (sRNA) in MM, sRNA sequencing was performed on exosomes from MM patients and healthy donors. Our data showed that piR-004800 was expressed at a higher level in MM compared to control samples. Moreover, the expression levels of piR-004800 and the ISS stages of MM were positively correlated.

In MM cells, we further demonstrated that downregulation of piR-004800 inhibited cell proliferation through induction of apoptosis and autophagic death; this outcome phenocopies the effect of FTY720 (Fingolimod) (22). FTY720 is a synthetic compound from fungal secondary metabolite myriocin (ISP-I). It is a new drug for multiple sclerosis and has potential for organ transplantation and cancer treatment (23). Preclinical assessment indicates that FTY720 possesses anti-cancer activity as a non-competitive S1PR inhibitor (24). Some studies showed that the S1PR signaling pathway regulates the expression of microRNAs, thereby contributing to the pathophysiological processes (25). Here, our study demonstrates the mutual effect between S1P and piR-004800. Inhibition of S1PRs by FTY720 downregulates the expression of piR-004800. This is recapitulated by the downregulation of CDC42, one of the downstream signaling pathways of G_i_ protein in MM (20). Conversely, activation of S1PRS by S1P upregulates piR-004800. These results indicated that S1PR signaling pathway regulates the expression of piR-004800 in MM cells.

S1P/S1PR/G-proteins could activate various signaling pathways in the cell including PI3K/Akt/mTOR (11). PI3K/Akt/mTOR pathway being activated by S1P could significantly inhibit the apoptotic cell death of granulosa cells (26). Our previous studies showed that S1P/FTY720 regulates PI3K/Akt/mTOR, thereby affecting apoptosis and autophagy in MM cells (15, 22). In this study, we showed that downregulation of piR-004800 decreases the expression of p-AKT and p-mTOR. Influencing the expression of certain tumor-related proteins through silencing mRNA similar to other non-coding small RNAs, piRNA can regulate the origination and development of tumor cells (6, 27). The exact mechanism of how piR-004800 regulates PI3K/AKT/mTOR deserves further investigation.

piRNAs have shown important roles in tumorgenesis and development. Our *in vivo* study showed that injection of antagomir-4800 into tumors in xenograft mice significantly inhibited tumor growth. These data highlight the possibility of piR-004800 as a potent molecular therapy target.

**Figure 7:**
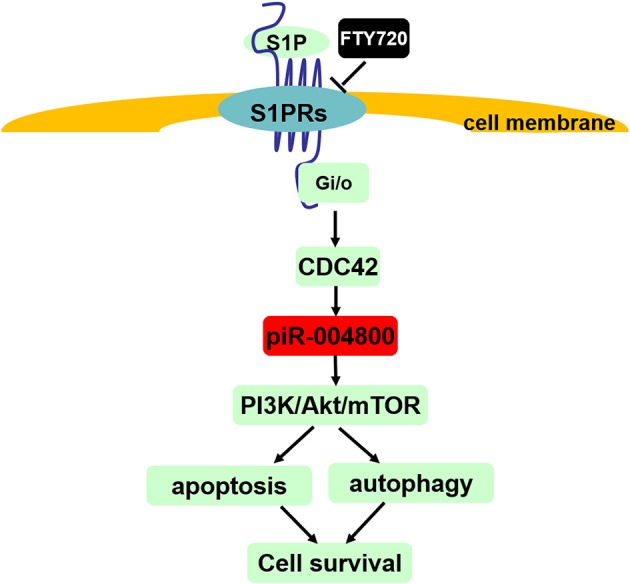
Summary of S1PR signaling pathway-mediated regulation of cell survival through piR-004800 in multiple myeloma cells. S1P binds to and activates S1P receptors (S1P1~S1P5) which all can couple to G_i/o_, and CDC42 is the downstream of G_i/o_. S1PR signaling pathway regulates PI3K/Akt/mTOR pathway through piR-004800 to affect apoptosis and autophagy in MM cells, by which to keep cell survival. FTY720 acts as a S1PRs' inhibitor.

In summary, our study showed that piR-004800 promotes MM cell survival, which regulated by S1PR signaling pathway (Figure 7). Its expression was associated with disease staging, which suggests that piR-004800 may be a marker for MM disease progression. Exploration of targeted intervention methods for piRNA expression may provide a breakthrough for tumor gene therapy.

## Data Availability Statement

The datasets generated for this study can be found in the NCBI Sequence Read Archive (https://www.ncbi.nlm.nih.gov/sra)(SRP239379).

## Ethics Statement

The studies involving human participants were reviewed and approved by the medical ethics committee of Shengjing Hospital. Written informed consent was obtained from all patients and healthy donors.

## Author Contributions

AL and HM conceived and designed the study. HM and FT performed the experiment and collected the data. HM and YZ performed the data analysis and statistical analysis. AL, HW, and ZL revised the manuscript critically. All authors commented on the draft of the paper and approved the final report.

### Conflict of Interest

The authors declare that the research was conducted in the absence of any commercial or financial relationships that could be construed as a potential conflict of interest.
