# Supplementary material for: PIWI-Interacting RNA-004800 Is Regulated by S1P Receptor Signaling Pathway to Keep Myeloma Cell Survival
Source: Front Oncol. 2020 Apr 15;10:438. doi: 10.3389/fonc.2020.00438 (PMC7175921; doi:10.3389/fonc.2020.00438)
Supplement: Supplementary file 1 [file Data_Sheet_1.PDF]

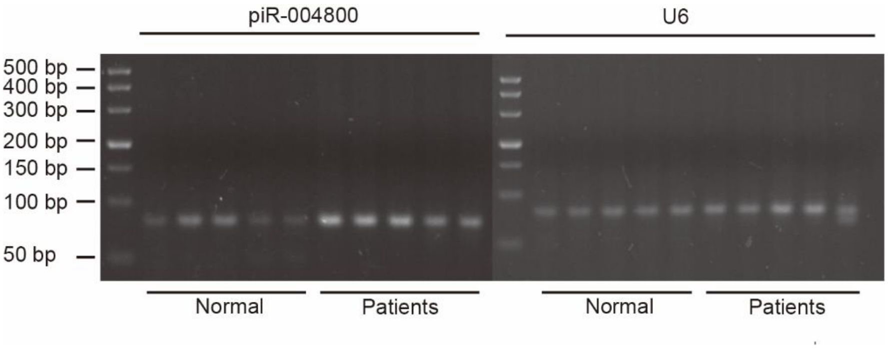

Supplemental Figure 1 **Expression and molecular weight of piR-004800 in the samples of MM patients and healthy controls**. Amplification products of piR-004800 by RT-PCR from 5 MM patients and 5 healthy controls were analyzed by agarose gel electrophoresis.

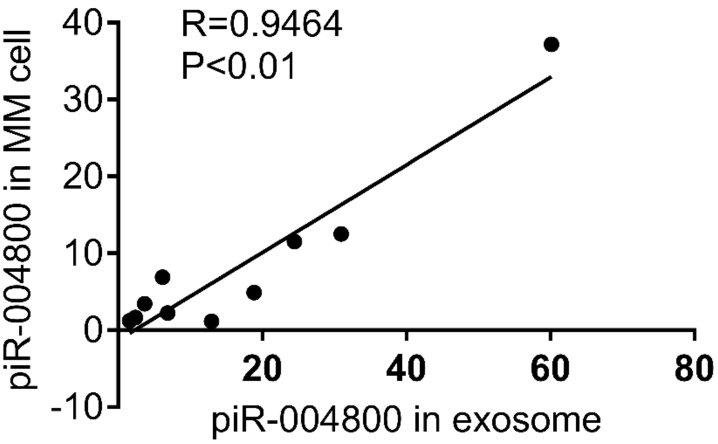

Supplemental Figure 2 **The correlation of piR-004800 levels between the exosomes and CD138$^+$ MM cells**. The expression of piR-004800 in the exosomes from bone marrow supernatant and CD138$^+$ primary MM cells of 10 MM patients was tested by qRT-PCR and subjected to a Spearman correlation analysis. R=correlation coefficient.

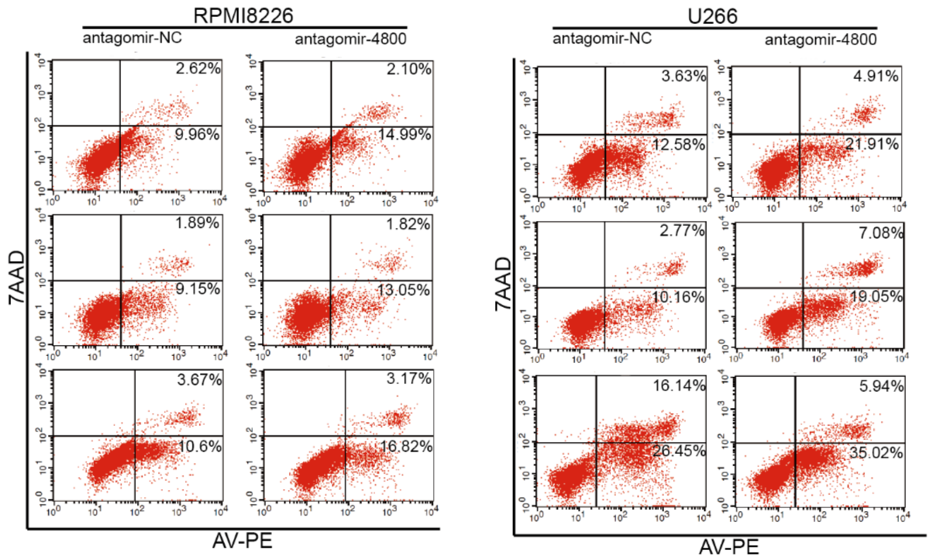

Supplemental Figure 3 **piR-004800 modulated apoptosis in MM cells.** RPMI8226 and U266 cells were transfected with antagomir-NC or antagomir-4800 and harvested after 48h, then stained with PE Annexin V/7-AAD and analyzed by flow cytometry. The experiments were repeated three times independently.

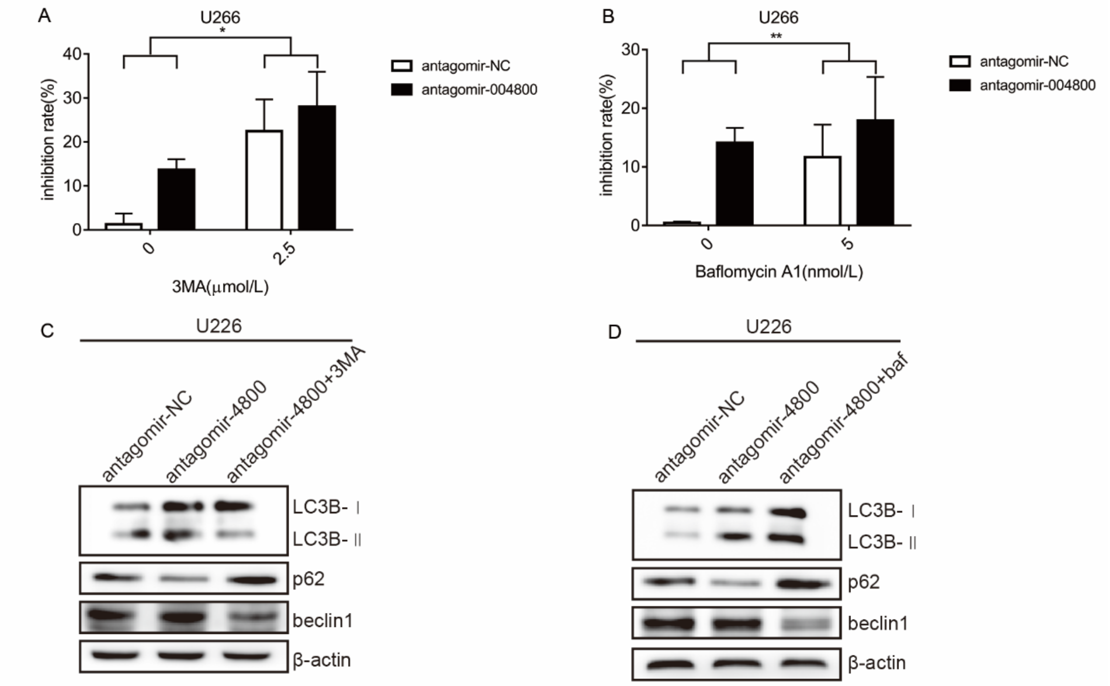

Supplemental Figure 4 **Autophagy inhibitors could rescue cell death induced by knockdown of piR-004800 in U266 cells.** (A, B) U266 cells were transfected with antagomir-NC or antagomir-4800, then 48h later the cells were treated with 3MA or Bafilomycin A1 for 24h, then cell inhibition rate was tested by MTS assays. (C, D) U266 cells were transfected with antagomir-NC or antagomir-4800, 48h later the cells were treated with 3MA or Bafilomycin A1 for 24h, then Western blot analysis was explored. *P<0.05, **P<0.01
